# Supplementary material for: HIVprotI: an integrated web based platform for prediction and design of HIV proteins inhibitors
Source: J Cheminform. 2018 Mar 9;10:12. doi: 10.1186/s13321-018-0266-y (PMC5845081; doi:10.1186/s13321-018-0266-y)
Supplement: Supplementary file 2 — Additional file 2. Source code of HIVProtI web server. [file 13321_2018_266_MOESM2_ESM.zip › HIVprotI_Source-code/hivprotI/weka-3-6-6/doc/weka/classifiers/IteratedSingleClassifierEnhancer.html]

IteratedSingleClassifierEnhancer


---


|  |  |  |
| --- | --- | --- |
| **Overview** \| **Package** \| **Class** \| **Tree** \| **Deprecated** \| **Index** \| **Help** \| **Weka's home** | |  |
| **PREV CLASS**   **NEXT CLASS** | **FRAMES**    **NO FRAMES**     **All Classes** |  |
| SUMMARY: NESTED \| FIELD \| CONSTR \| METHOD | DETAIL: FIELD \| CONSTR \| METHOD |  |


---


## weka.classifiers Class IteratedSingleClassifierEnhancer

```
java.lang.Object
  weka.classifiers.Classifier
      weka.classifiers.SingleClassifierEnhancer
          weka.classifiers.IteratedSingleClassifierEnhancer
```

**All Implemented Interfaces:**: java.io.Serializable, java.lang.Cloneable, CapabilitiesHandler, OptionHandler, RevisionHandler

**Direct Known Subclasses:**: AdditiveRegression, RandomizableIteratedSingleClassifierEnhancer

---

``` public abstract class IteratedSingleClassifierEnhancer extends SingleClassifierEnhancer ```

Abstract utility class for handling settings common to
meta classifiers that build an ensemble from a single base learner.

**Version:**
:   $Revision: 1.4 $

**Author:**
:   Eibe Frank (eibe@cs.waikato.ac.nz)

**See Also:**: Serialized Form

---

**Constructor Summary**

`IteratedSingleClassifierEnhancer()`


| **Method Summary** | |
| --- | --- |
| `void` | `buildClassifier(Instances data)`             Stump method for building the classifiers. |
| `int` | `getNumIterations()`             Gets the number of bagging iterations |
| `java.lang.String[]` | `getOptions()`             Gets the current settings of the classifier. |
| `java.util.Enumeration` | `listOptions()`             Returns an enumeration describing the available options. |
| `java.lang.String` | `numIterationsTipText()`             Returns the tip text for this property |
| `void` | `setNumIterations(int numIterations)`             Sets the number of bagging iterations |
| `void` | `setOptions(java.lang.String[] options)`             Parses a given list of options. |

**Methods inherited from class weka.classifiers.SingleClassifierEnhancer**

`classifierTipText, getCapabilities, getClassifier, setClassifier`

**Methods inherited from class weka.classifiers.Classifier**

`classifyInstance, debugTipText, distributionForInstance, forName, getDebug, getRevision, makeCopies, makeCopy, setDebug`

**Methods inherited from class java.lang.Object**

`equals, getClass, hashCode, notify, notifyAll, toString, wait, wait, wait`

**Constructor Detail**

### IteratedSingleClassifierEnhancer

```
public IteratedSingleClassifierEnhancer()
```


**Method Detail**

### buildClassifier

```
public void buildClassifier(Instances data)
                     throws java.lang.Exception
```

:   Stump method for building the classifiers.

    :   **Specified by:**: `buildClassifier` in class `Classifier`
    :   **Parameters:**: `data` - the training data to be used for generating the bagged classifier. **Throws:**: `java.lang.Exception` - if the classifier could not be built successfully

---


### listOptions

```
public java.util.Enumeration listOptions()
```

:   Returns an enumeration describing the available options.

    :   **Specified by:**: `listOptions` in interface `OptionHandler` **Overrides:**: `listOptions` in class `SingleClassifierEnhancer`
    :   **Returns:**: an enumeration of all the available options.

---


### setOptions

```
public void setOptions(java.lang.String[] options)
                throws java.lang.Exception
```

:   Parses a given list of options. Valid options are:

    -W classname   
    Specify the full class name of the base learner.

    -I num   
    Set the number of iterations (default 10).

    Options after -- are passed to the designated classifier.

    :   **Specified by:**: `setOptions` in interface `OptionHandler` **Overrides:**: `setOptions` in class `SingleClassifierEnhancer`
    :   **Parameters:**: `options` - the list of options as an array of strings **Throws:**: `java.lang.Exception` - if an option is not supported

---


### getOptions

```
public java.lang.String[] getOptions()
```

:   Gets the current settings of the classifier.

    :   **Specified by:**: `getOptions` in interface `OptionHandler` **Overrides:**: `getOptions` in class `SingleClassifierEnhancer`
    :   **Returns:**: an array of strings suitable for passing to setOptions

---


### numIterationsTipText

```
public java.lang.String numIterationsTipText()
```

:   Returns the tip text for this property

    :   **Returns:**: tip text for this property suitable for displaying in the explorer/experimenter gui

---


### setNumIterations

```
public void setNumIterations(int numIterations)
```

:   Sets the number of bagging iterations

---


### getNumIterations

```
public int getNumIterations()
```

:   Gets the number of bagging iterations

    :   **Returns:**: the maximum number of bagging iterations


---


|  |  |  |
| --- | --- | --- |
| **Overview** \| **Package** \| **Class** \| **Tree** \| **Deprecated** \| **Index** \| **Help** \| **Weka's home** | |  |
| **PREV CLASS**   **NEXT CLASS** | **FRAMES**    **NO FRAMES**     **All Classes** |  |
| SUMMARY: NESTED \| FIELD \| CONSTR \| METHOD | DETAIL: FIELD \| CONSTR \| METHOD |  |


---
